# Factors Associated With Disparities in Hospital Readmission Rates Among US Adults Dually Eligible for Medicare and Medicaid

**DOI:** 10.1001/jamahealthforum.2021.4611

**Published:** 2022-01-28

**Authors:** David Silvestri, Demetri Goutos, Anouk Lloren, Sheng Zhou, Guohai Zhou, Thalia Farietta, Sana Charania, Jeph Herrin, Alon Peltz, Zhenqiu Lin, Susannah Bernheim

**Affiliations:** 1 National Clinician Scholars Program, Yale School of Medicine, New Haven, Connecticut; 2 Department of Emergency Medicine, Yale School of Medicine, New Haven, Connecticut; 3 The Yale Center for Outcomes Research and Evaluation, Yale New Haven Health Services Corporation, New Haven, Connecticut; 4 Mathematica Policy Research, Cambridge, Massachusetts; 5 Department of Medicine, Brigham and Women’s Hospital, Boston, Massachusetts; 6 Vertex Pharmaceuticals Incorporated, Boston, Massachusetts; 7 Department of Health Policy and Management, Milken Institute School of Public Health, George Washington University, Washington, DC; 8 Section of Cardiovascular Medicine, Department of Internal Medicine, Yale School of Medicine, New Haven, Connecticut; 9 Flying Buttress Associates, Charlottesville, Virginia; 10 Department of Population Medicine, Harvard Medical School, Harvard Pilgrim Health Care Institute, Boston, Massachusetts; 11 Department of Internal Medicine, Yale School of Medicine, New Haven, Connecticut

## Abstract

**Importance:**

Low-income older adults who are dually eligible (DE) for Medicare and Medicaid often experience worse outcomes following hospitalization. Among other federal policies aimed at improving health for DE patients, Medicare has recently begun reporting disparities in within-hospital readmissions. The degree to which disparities for DE patients are owing to differences in community-level factors or, conversely, are amenable to hospital quality improvement, remains heavily debated.

**Objective:**

To examine the extent to which within-hospital disparities in 30-day readmission rates for DE patients are ameliorated by state- and community-level factors.

**Design, Setting, and Participants:**

In this retrospective cohort study, Centers for Medicare & Medicaid Services (CMS) Disparity Methods were used to calculate within-hospital disparities in 30-day risk-adjusted readmission rates for DE vs non-DE patients in US hospitals participating in Medicare. All analyses were performed in February and March 2019. The study included Medicare patients (aged ≥65 years) hospitalized for acute myocardial infarction (AMI), heart failure (HF), or pneumonia in 2014 to 2017.

**Main Outcomes and Measures:**

Within-hospital disparities, as measured by the rate difference (RD) in 30-day readmission between DE vs non-DE patients following admission for AMI, HF, or pneumonia; variance across hospitals; and correlation of hospital RDs with and without adjustment for state Medicaid eligibility policies and community-level factors.

**Results:**

The final sample included 475 444 patients admitted for AMI, 898 395 for HF, and 1 214 282 for pneumonia, of whom 13.2%, 17.4%, and 23.0% were DE patients, respectively. Dually eligible patients had higher 30-day readmission rates relative to non-DE patients (RD >0) in 99.0% (AMI), 99.4% (HF), and 97.5% (pneumonia) of US hospitals. Across hospitals, the mean (IQR) RD between DE vs non-DE was 1.00% (0.87%-1.10%) for AMI, 0.82% (0.73%-0.96%) for HF, and 0.53% (0.37%-0.71%) for pneumonia. The mean (IQR) RD after adjustment for community-level factors was 0.87% (0.73%-0.97%) for AMI, 0.67% (0.57%-0.80%) for HF, and 0.42% (0.29%-0.57%) for pneumonia. Relative hospital rankings of corresponding within-hospital disparities before and after community-level adjustment were highly correlated (Pearson coefficient, 0.98).

**Conclusions and Relevance:**

In this cohort study, within-hospital disparities in 30-day readmission for DE patients were modestly associated with differences in state Medicaid policies and community-level factors. This suggests that remaining variation in these disparities should be the focus of hospital efforts to improve the quality of care transitions at discharge for DE patients in efforts to advance equity.

## Key Points

**Question:**

To what extent are state- and community-level factors associated with within-hospital disparities in hospital readmission for dual-eligible Medicare patients?

**Findings:**

In this cohort study of 2.5 million US adults aged 65 years or older, within**-**hospital disparities in 30-day readmission for dual-eligible patients persisted after accounting for state- and community-level social and health service availability factors. There was no meaningful change in hospital ranking or between hospital variation in disparity performance when adjustments for community-level factors were included.

**Meaning:**

Hospital disparities in 30-day readmission rates for dual-eligible patients are not fully explained by differences in community-level factors, and this persistent variation suggests continued opportunities for hospital efforts to advance equity in health care outcomes.

## Teaser

This cohort study examines the extent to which state- and community-level factors are associated with within-hospital disparities in hospital readmission rates for dual-eligible Medicare patients.

## Introduction

Low-income older US adults who are dually eligible (DE) for both Medicare and Medicaid insurance often experience worse outcomes and higher rates of readmission following hospitalization.^1^ In recent years, numerous government programs and policies have aimed to mitigate these inequities, including by enhancing integration of medical and social resources aimed to improve outcomes for DE adults.^2,3^ Starting in 2018, the Centers for Medicare & Medicaid Services (CMS) began reporting disparities in readmission rates for DE patients, with all US hospitals now receiving confidential reports of their hospital-specific disparities in condition-specific 30-day risk-adjusted readmission rates for DE patients.^4,5^

The CMS hospital-specific disparity measures account for potential differences in clinical risk factors across patient populations and attribute unexplained variation in outcomes after clinical risk adjustment to differences in hospital performance for DE patients. In turn, variation across hospitals in these hospital-specific disparities is taken to reflect differences in hospitals’ relative performance in achieving equity for DE patients. Although it is possible for such variation within and across hospitals to reflect differences in hospital quality or care delivery processes, they might also stem from patient- and community-level factors seemingly beyond hospitals’ immediate control. Community-level measures of social risk may mediate observed disparities by virtue of their community-level effects and reflection of individual-level risks borne by residents of the community. Social assists of a community, such as affordable housing and economic opportunity, vary widely across US neighborhoods, and these social factors may strongly affect outcomes following hospitalization.^6,7^ Studies suggest that hospitals serving patients from more socioeconomically disadvantaged communities experience greater difficulty achieving comparable readmission rates for Medicare patients as other hospitals,^8,9,10,11^ although the strength of this finding has been questioned.^12^ In addition, because Medicaid income eligibility standards vary by state there are concerns that this variation manifests in different levels of unmeasured social risk in hospitals in some geographies relative to others.^13^ Finally, regional differences in local health service availability, especially including local primary care capacity, have been associated with differences in hospital readmission rates for all patients,^14,15^ but may reflect regional or market attributes outside local hospital control.

With increasing focus on hospital disparities in outcomes for DE patients, the extent to which social risk and resources measured at state- and community-levels explain these reported disparities, and differences in those disparities across hospitals, is not well established. Understanding whether and how disparities for DE patients within hospitals persist when accounting for these factors can inform federal policy making and quality improvement investments to advance equity for socioeconomically disadvantaged populations. Therefore, we sought to evaluate the effect of accounting for community-level factors on within-hospital disparities in readmission between DE and non-DE patients following admission for each of 3 common conditions among Medicare patients: acute myocardial infarction (AMI), heart failure (HF), and pneumonia. We applied standard CMS disparity methods, accounting for clinical risk differences across hospitals, and included community-level factors across 3 domains: social factors, state policies, and health services availability. For each condition, we assessed the change in magnitude and variation across hospitals in the degree of within-hospital disparity for DE patients, with and without inclusion of each domain of community-level factors, and describe the correlation in relative hospital performance ranking with and without community-level adjustment.

## Methods

### Data Sources

We used Medicare Part A and B Standard Analytic Files and Enrollment Files to identify the study cohort, clinical risk factors for adjustment, and enrollment information including DE status and zip code of residence. We obtained community-level factors from the US Census Bureau’s American Community Survey (ACS).^16^ We used publicly available data to categorize state DE enrollment and eligibility policies,^17,18^ and data from the Health Resources and Services Administration (HRSA) Area Health Resources Files (AHRF) to categorize availability of local health services.^19^ We used the American Hospital Association (AHA) Annual Survey to identify hospital characteristics.^20^

This report follows Strengthening the Reporting of Observational Studies in Epidemiology (STROBE) reporting guidelines for cohort studies. Research approval was obtained by the Yale University institutional review board, who granted exemption from obtaining patient consent as all patient data are deidentified.

### Study Design and Population

We conducted a retrospective cohort study of Medicare fee-for-service patients aged 65 years and older admitted to US acute care, critical access, and Veterans Affairs hospitals with a principal discharge diagnosis of AMI, HF, or pneumonia from July 1, 2014, to June 30, 2017. Cohort selection criteria followed publicly reported measure specifications used by CMS; we further divided the cohort by DE status, defined as eligibility for full state Medicaid benefits.^1,21^ We included multiple admissions per patient if they were separated by at least 30 days. To ensure adequate data for clinical risk adjustment, we included only admissions for which patients were continuously enrolled in Medicare for at least 12 months prior to the index admission. Similarly, to ensure accurate identification of readmissions, we included only admissions with at least 30 days of enrollment after discharge. We excluded any admission ending with in-hospital death or discharge against medical advice. We attributed admissions resulting in transfer between hospitals to the hospital that ultimately discharged the patient.

### Outcome

For each of the 3 conditions studied, our primary outcome was the difference in 30-day risk-adjusted readmission rate between DE and non-DE patients admitted to the same hospital (hereafter: within-hospital disparity) using standard CMS methods.^4^ Briefly, this approach computes for each hospital the risk-adjusted readmission rates experienced by DE and non-DE patients using hierarchical logistic regression, which aligns with currently implemented risk-standardized performance measures as previously described.^21^ In the model, the effect of being DE on readmission risk specific to each hospital is calculated using a random effect. The model accounts for the hospital proportion of patients in the cohort who are DE. Each hospital’s within-hospital disparity is calculated by CMS as a rate difference (RD), which is the difference in the predicted probability of readmission between a DE patient and a non-DE patient with the same average mix of clinical risk factors at that hospital. The RD is reported as a percentage.

### Domain 1: State Medicaid Eligibility Policies

We categorized states by their Medicaid eligibility and enrollment processes for older adults (aged ≥65 years). We used publicly available state Medicaid eligibility levels for the annual household income and assets limits for 3 eligibility groups: *categorically eligible, poverty level*, and *medically needy pathways*.^17,18,22^ We identified these 3 groups based on an analysis of Medicaid Enrollment data which identified that these eligibility groups accounted for most (>80%) DE patients (eAppendix A in the Supplement). We accounted for enrollment differences based on whether the state uses a single federal application and federal criteria, a separate state application and federal criteria, or combination state-specific application and criteria. State assignment was based on the patient’s home address (and not the hospital address).

### Domain 2: Health Service Availability

To account for differences in health service availability on within-hospital disparities in readmission across settings, we used the AHRF to delineate primary care density—the number of primary care clinicians (general practitioners, family medicine practitioners, general internal medicine practitioners, physician assistants, nurse practitioners, and clinical nurse specialists) per 100 000 population—for all US counties.^14^ We classified primary care density into quintiles at the county level, linked to Medicare patients for analysis.

### Domain 3: Social Factors

We employed the National Academy of Medicine’s conceptual framework for the influence of social risk factors on health care outcomes to identify indicators of social risk across the following 5 subdomains: socioeconomic position; race, ethnicity, and cultural context; gender; social relationships; and residential and community context.^6^ Patient sex is presently accounted for in the CMS risk-adjustment model for these 3 measures,^21^ although this may imperfectly approximate gender. For the remaining 4 subdomains, we identified corresponding indicators from the American Community Survey (Table 1). For each, we used American Community Survey 5-year estimates at the zip Code Tabulation Area (ZCTA) level, classifying ZCTAs by quintiles and dichotomizing into the highest-risk vs remaining four quintiles, then linking to Medicare patients’ residence zip code for analysis. This approach supports an examination of the effect of the patient’s social environment, as opposed to that of the hospital, and parallels approaches performed elsewhere.^15,23,24^ We excluded less than 1% of the cohort (0.60%, 0.57%, and 0.65%, for AMI, HF, and pneumonia, respectively) owing to missing social risk factor data. Race estimates in the American Community Survey are obtained via patient self-report, captured through the Census.

**Table 1.** Community-Level Indicators of Social Risk Used for Within-Hospital Disparity Adjustment^a^

National Academies of Medicine risk domain	Community-level indicator	Detailed definition
Socioeconomic position	Socioeconomic status score	AHRQ socioeconomic score: composite weighted score consisting of: Median income in past 12 months Median value of owner-occupied housing unit Percent of persons below federally defined poverty line Percent of persons aged 16 years or older in civilian labor force who are unemployed and actively seeking work Percent of persons aged 25 years or older with at least four years of college Percent of persons aged 25 years or older with less than a 12th grade education Percent of area housing units with >1 occupant per room
Race, ethnicity, and cultural context	Black race	Percent of persons who are Black or African American alone
	Hispanic ethnicity	Percent of persons who are Hispanic or Latino
	Limited English proficiency	Percent of persons aged 5 years or older who do not speak English at home and who speak English less than “very well”
	Foreign born nativity	Percent of persons who are foreign born
Social relationships	Unmarried or spouse absent	Percent of persons aged 15 years or older who are unmarried or married with spouse absent
	Living without family	Percent of persons aged 65 years or older who live in nonfamily households or group quarters
Residential and community context	Poor vehicular availability	Percent of households with no vehicle available
	Vacant housing	Percent of housing units that are vacant
	Food or cash assistance	Percent of households receiving public assistance income or food stamps/SNAP in the past 12 months

^a^ All indicators are obtained from American Community Survey (ACS) 2017 5-year estimates (with exception to the Agency for Healthcare Research and Quality [AHRQ] socioeconomic status score, obtained from ACS 2013 5-year estimates), measured at the zip Code Tabulation Area.

### Statistical Analysis

First, for each condition cohort we summarized unadjusted readmission rates for DE and non-DE patients. We compared the distribution of community-level factors between DE and non-DE patients using χ^2^ tests to test for differences between groups. For each condition (AMI, HF, and pneumonia) we then calculated hospital RD between DE and non-DE patients using 4 separate models. The first model used the standard CMS disparities method (model 1).^1,4^ Briefly, this is a mixed effects logistic regression model with patient clinical risk factors, an indicator for DE status, a random intercept and a random slope for the DE effect. The random slope captures the difference in readmission risk between DE and non-DE patients; this parameter, along with the random intercept, is used to estimate for each hospital the difference in readmission rate (rate difference) for 2 average patients who differ only in DE status for that hospital (eAppendices A and C in the Supplement). We then estimated 3 extensions of this model by sequentially adding: state Medicaid policy differences (model 2); health service availability (model 3); and the full set of social factors (model 4). We recalculated the RD using each model. For each model we report the variance in the DE effect across hospitals and compared the distributions to those from the original model using paired *t* tests and Pearson correlation. Finally, within each condition-specific cohort, we identified the top 5% of hospitals with most changes in disparity score, after adjusting for community-factors, and used bivariable statistics to compare their characteristics to all other hospitals. All analyses were conducted with SAS statistical software (version 9.4; SAS Institute, Inc), with statistical significance set at *P* < .05. Analyses were performed in February and March 2019.

## Results

The final sample included 475 444 Medicare patients admitted for AMI, accounting for 507 219 index admissions to 4188 hospitals; 898 395 patients admitted for HF, accounting for 1 214 282 index admissions to 4697 hospitals; and 1 209 845 patients admitted for pneumonia, accounting for 1 419 412 index admissions to 4749 hospitals. Dually eligible patients comprised 13.2% of patients hospitalized for AMI (n = 66 201 index admissions), 17.4% for HF (n = 208 311 index admissions), and 23.0% for pneumonia (n = 321 506 index admissions). Median hospital unadjusted readmission rates were higher for DE patients vs non-DE patients for AMI (16.7%; IQR, 0.0%-26.2% vs 14.5%; IQR, 6.7%-20.0%; HF, 22.9%; IQR, 15.0%-29.2% vs 20.3%; IQR, 16.7%-24.0%, and pneumonia, 17.0%; IQR, 11.1%-21.7% vs 15.1%, IQR, 11.9%-18.0%).

### Social Factors

Social factors differed between DE patients and non-DE patients across most conditions (Table 2). Compared with non-DE patients, a significantly greater share of DE patients lived in the US communities with the highest proportions of limited English speakers (range across condition cohorts: 40.7%-45.2% vs 29.1%-30.1%); non-US born (41.1%-45.6% vs 32.5%-34.9%), Black residents (28.6%-34.9% vs 22.7%-27.1%), or Hispanic residents (32.4%-36.6% vs 22.3%-23.2%); and single-adult (33.0%-39.4% vs 19.4%-22.8%) or single-individual (24.0%-25.1% vs 17.1%-18.4%) households. Similarly, DE patients also were more likely to reside in those US communities with lowest vehicular availability (37.1%-42.9% vs 22.7%-25.7%) and poorest overall socioeconomic status (28.2%-32.0% vs 13.1%-14.8%) (all *P* values <.001).

**Table 2.** Distribution of Community-Level Indicators of Social Risk Among Dual-Eligible and All Other Medicare Patients

Community-level indicators of social risk	AMI			HF			Pneumonia		
	Prevalence, %^a^			Prevalence, %^a^			Prevalence, %^a^		
	Total	Dual eligible	Nondual eligible	Total	Dual eligible	Nondual eligible	Total	Dual eligible	Nondual eligible
Socioeconomic position									
Low composite SES score^b^	20.8	32.5	13.6	22.6	32.5	14.8	21.2	28.2	13.1
Race, ethnicity, cultural context									
Black race^b^	30.0	30.0	23.0	34.7	34.9	27.1	30.2	28.6	22.7
Hispanic ethnicity^b^	28.8	36.6	22.9	29.6	35.7	23.2	29.2	32.4	22.3
Limited English proficiency^b^	31.1	45.1	29.1	33.2	45.2	30.1	32.0	40.7	29.6
Non-US born^b^	34.0	45.2	32.5	36.6	45.6	34.9	35.2	41.1	33.7
Social relationships									
Unmarried or spouse absent^b^	22.1	37.1	19.9	25.7	39.4	22.8	22.5	33.0	19.4
Living without family^b^	18.0	24.5	17.1	19.5	25.1	18.4	19.0	24.0	17.5
Residential/community context									
Poor vehicular availability^b^	25.6	41.3	23.2	28.6	42.9	25.7	26.0	37.1	22.7
Vacant housing^b^	7.5	6.5	7.7	6.3	5.6	6.5	6.6	5.9	6.8
Food or cash assistance^b^	20.2	23.2	24.7	21.9	23.5	25.2	20.0	23.9	24.2

Abbreviations: AMI, acute myocardial infarction; HF, heart failure; SES, socioeconomic status.

^a^ Prevalence is reported among total dual eligible and all other patients respectively in each condition-specific cohort (AMI: 61 204 dual eligible, 414 240 all other patients; HF: 152 355 dual eligible, 746 040 all other patients; pneumonia: 266 564 dual eligible, 943 281 all other patients). *P* values for nondichotomous ordinal (ie, quintiles) categorical variables were computed using the Mann-Whitney U-test for comparison using α = 0.05. All *P* < .001 except the food or cash assistance variable for patients with pneumonia (*P* = .03).

^b^ Variables indicate beneficiary residence in the zip Code Tabulation Area with the highest-risk quintile in terms of the Agency for Healthcare Research and Quality Socioeconomic Status Index composite score (lowest), Black race (highest), Hispanic origin (highest), percent of population 5 years or older who do not speak English at home and who speak English less than “very well” (highest), percent of population who are non-US born (highest), percent of population aged 15 years or older who are unmarried or married with spouse absent (highest), percent of population aged 65 years or older who live in nonfamily or group homes (highest), percent of households with no vehicular availability (highest), percent of housing units that are vacant (highest), or percent of population receiving food or cash assistance (highest). Reference is the remaining 4 quintiles.

### Within-Hospital Disparities

**Figure 1.**
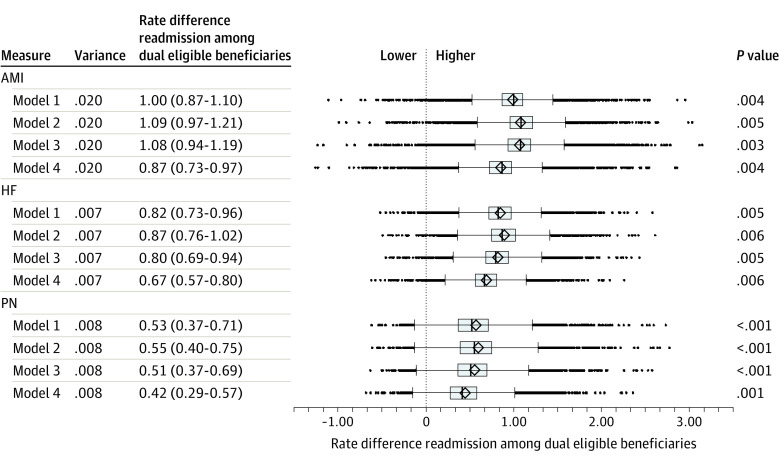
Within-Hospital Disparities in Risk-Standardized Readmission Rates Before and After Adjustment for Community-Level Indicators of Social Risk AMI Indicates acute myocardial infarction; HF, heart failure; PN, pneumonia. Model 1 reflects the standard CMS approach. Model 2 adjusts the standard Centers for Medicare & Medicaid Services (CMS) approach for state Medicaid policy differences pertaining to eligibility and enrollment. Model 3 adjusts the standard CMS approach for state Medicaid policy differences pertaining to eligibility and enrollment, as well as health services availability; model 4 adjusts the standard CMS approach for state Medicaid policy differences pertaining to eligibility and enrollment, health services availability, as well as community-level social risk factors.

At most hospitals, risk-adjusted readmission rates were higher for DE than for non-DE patients (Figure 1, model 1). The RD in readmission between DE and non-DE patients was greater than 0 for 98.9%, 99.4%, and 97.4% of hospitals, for AMI, HF, and pneumonia admissions, respectively. The median RD was 1.00% (IQR, 0.87%-1.10%) for AMI, 0.82% (IQR, 0.73%-0.96%) for HF, and 0.53% (IQR, 0.37%-0.71%) for pneumonia. Adjustment for state policies (model 2), health service availability (model 3), and social factors (model 4) each significantly affected RDs compared with the unadjusted model. Adjustment for all factors narrowed the overall magnitude of within-hospital RD between DE patients and non-DE patients for all 3 conditions (Figure 1, model 4; all *P* < .001). The median RD after adjustment for all community-level factors was 0.87% (IQR, 0.73%-0.97%) for AMI, 0.67% (IQR, 0.57%-0.80%) for HF, and 0.42% (IQR, 0.29%-0.57%) for pneumonia.

**Figure 2.**
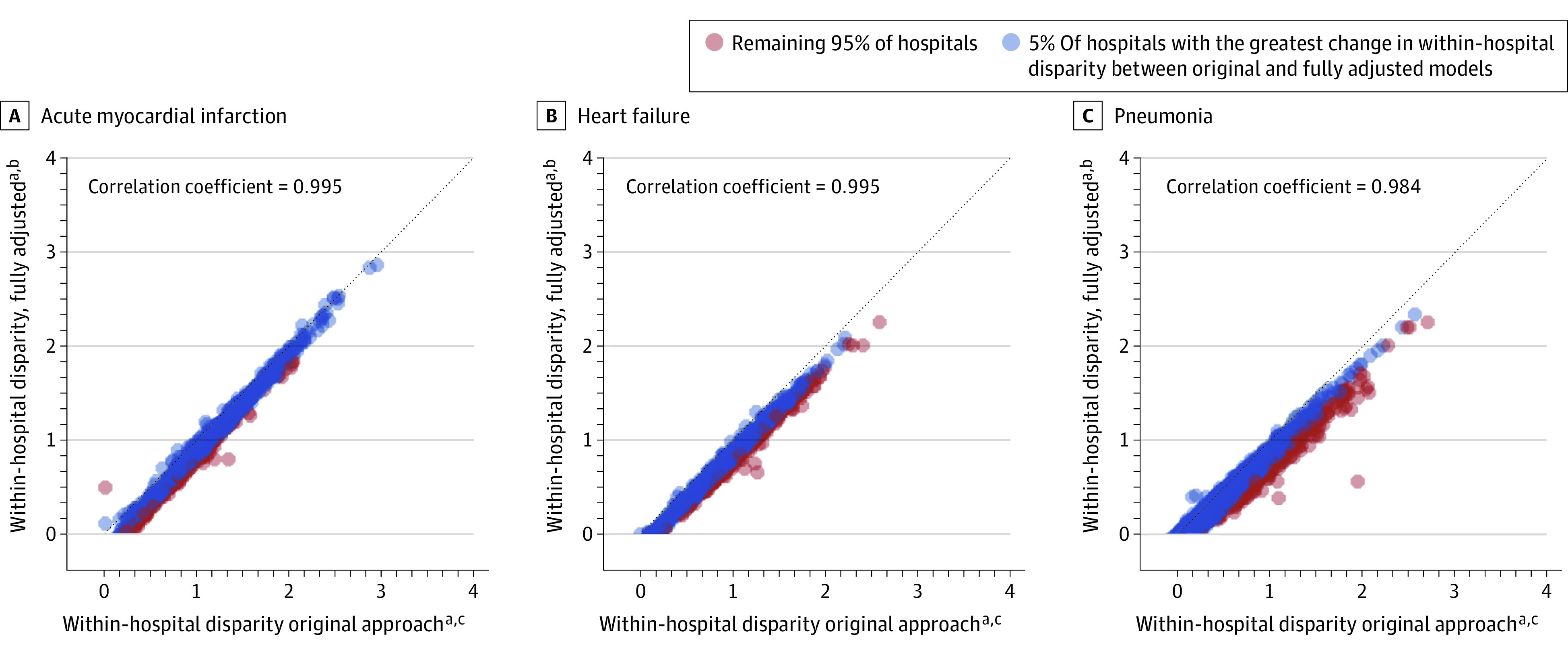
Correlation in Within-Hospital Disparities in Risk-Standardized Readmission Rates Before and After Adjustment for Community-Level Indicators of Social Risk ^a^Within-hospital disparity reflects the difference in risk-standardized readmission rates between dual-eligible and all other patients. ^b^Fully-adjusted within-hospital disparities accounts for clinical risks (age, sex, clinical conditions), as well as state Medicaid policies, local health service availability, and community-level indicators of social risk. ^c^Original approach to measuring within-hospital disparities accounts for differences in clinical risks (age, sex, clinical conditions).

For all 3 conditions, there was significant variation across hospitals in the degree of within-hospital readmission disparities between DE and non-DE patients (Figure 1, model 1; AMI, *P* = .005; HF, *P* = .005; PN, *P* < .001). Adjusting for community factors did not affect this overall variation (Figure 1, models 2-4). The relative rankings of hospital-specific RDs were similar with and without adjustment (Figure 2). Pearson correlation coefficient comparing model 1 and model 4 hospital RDs were high for all 3 conditions (AMI, 0.99; HF, 0.99; pneumonia, 0.98); correlations between models 2 and 3 were similar. Compared with all other hospitals, the 5 percent of hospitals whose magnitude of RD was most affected by adjustment for community-level factors were more often larger, urban, academic teaching hospitals, but were less often safety-net hospitals (Table 3).

**Table 3.** Characteristics of Hospitals With the Greatest Change in Within-Hospital Disparities Before and After Adjusting for Community-Level Indicators of Social Risk

Characteristic	Acute myocardial infarction			Heart failure			Pneumonia		
	Hospitals with change in disparity^a^		*P* value^b^	Hospitals with change in disparity^a^		*P* value^b^	Hospitals with change in disparity^a^		*P* value^b^
	High	Low		High	Low		High	Low	
Condition-related characteristics									
Total No. of hospitalizations for condition, No. (IQR)^c^	245 (160-381)	26 (5-149)	<.001	564 (247-920)	110 (33-345)	<.001	437 (251-683)	169 (63-408)	<.001
Dual-eligible share of condition hospitalizations, median (IQR), %^c^	12.0 (7.1-21.8)	13.0 (5.6-27.9)	<.001	20.5 (13.8-34)	16.3 (9.7-25.4)	<.001	26.2 (16.6-39.3)	21.7 (14.6-31.5)	<.001
Condition-specific overall RSRR under HRRP, median (IQR), %	17.3 (16.4-18.1)	16.0 (15.7-16.3)	<.001	21.8 (20.5-23.3)	21.6 (20.9-22.5)	<.001	19.3 (18.2-20.2)	16.6 (15.9-17.3)	<.001
General characteristics									
Total bed capacity, %									
<300	54.2	82.2	<.001	38.1	85.2	<.001	49.0	84.5	.08
300-600	31.1	13.8		33.2	12.0		34.2	12.0	
>600	14.7	4.0		28.8	2.3		16.8	3.4	
Ownership, %									
Public (government)	7.9	21.1	.001	12.4	23.6	<.001	12.2	23.7	.03
Private not-for-profit	71.2	62.4		73.9	59.8		63.8	60.0	
Private for-profit	20.1	16.5		13.7	16.7		24.0	16.3	
Teaching status, %									
Council of Teaching Hospitals	20.3	5.2	<.001	26.1	4.2	<.001	18.4	4.7	.008
Teaching, non-COTH	41.2	24.3		46.0	22.0		40.3	22.3	
Non-teaching	38.4	70.5		27.9	73.8		41.3	73.0	
Urban, %^d^	91.0	59.5	<.001	89.4	56.2	<.001	90.3	56.4	.92
Safety-net hospital, %	15.8	28.7	<.001	21.7	31.1	<.001	22.5	31.1	.007
Critical access hospital, %	1.0	24.6	<.001	2.6	29.8	<.001	0	29.9	<.001
Characteristics of disparity change									
Size of change in within-hospital disparity rate after adjustment, median absolute rate change (IQR), %^e^	0.21 (0.20-0.23)	0.13 (0.12-0.15)	<.001	0.23 (0.22-0.24)	0.16 (0.15-0.17)	<.001	0.30 (0.27-0.35)	0.10 (0.07-0.13)	<.001
Change in within-hospital disparity ranking by 2 or more deciles after adjustment, %	6.7	1.0		13.6	0.52		19.0	2.1	

Abbreviations: CMS, Centers for Medicare & Medicaid Services; COTH, Council of Teaching Hospitals; HRRP, Hospital Readmission Reduction Program; RSRR, risk-standardized readmission rate.

^a^ Within each condition-specific cohort, hospitals with high changes in disparities are the top 5% of hospitals in terms of the change in within-hospital disparities after adjusting for state-level dual eligibility policies, county-level health service availability, and community-level indicators of social risk. Hospitals with low changes in disparities reflect the remaining 95%.

^b^ *P* values for categorical variables were computed using the χ^2^ test for comparison using α = 0.05. *P* values for nonparametric variables (ie, median hospital values) are computed using the Kruskal-Wallis test for comparison using α = 0.05.

^c^ Total number of hospitalizations and the dual-eligible share of hospitalizations are for each condition.

^d^ Urban hospitals are those within Metropolitan Statistical Area, in reference to all other areas combined (micropolitan, rural).

^e^ Reflects magnitude (absolute value) of change in within-hospital disparity after adjustment for state Medicaid policies, local health service availability, and community-level indicators of social risk (compared with original CMS approach).

## Discussion

These findings demonstrate that disparities in 30-day hospital readmission rates for DE patients within the same hospital persist even after accounting for state- and community-level social factors. Although adjusting for state Medicaid eligibility policies, primary care availability, and social factors modestly attenuated the magnitude of within-hospital disparities for patients hospitalized for 3 common conditions (AMI, HF, and pneumonia), adjustment did not affect the overall significance of these disparities, nor the degree and ranking of variation in disparities across hospitals. Findings suggest that differences in inequities in hospital readmission rates for DE patients are not the primary result of differences measurable across communities, highlighting that hospitals may have a distinct role in advancing equity for socioeconomically disadvantaged patients.^25^

Although previous studies have examined the effect of community-level factors on patient outcomes,^26,27^ to our knowledge this is the first examination of such factors on within-hospital disparities in outcomes. Examining disparities between patient groups within individual hospitals—as opposed to across a large number of hospitals—increases the likelihood that identified disparities between patient groups reflect differences in care delivery processes or quality at a given particular hospital,^7^ rather than differential utilization of higher- or lower-quality hospitals by 1 patient group compared to the other. Moreover, by measuring within-hospital disparities using standard CMS methods currently in use for confidential reporting,^4^ our study augments prior literature,^23,28,29^ providing immediate relevance to current CMS disparity measure reporting.

Our analysis accounts for a uniquely broad set of state- and community-level factors to ensure that any variation in within-hospital disparities was not attributable to systematic differences in hospitals’ respective DE or non-DE populations or the community-level resources available to them. Our analysis controlled for the domains of social risk reported by the National Academies of Medicine,^6^ in addition to potential policy-mediated differences between DE populations across states. Because states differ in their approaches to Medicaid coverage—and thereby DE definitions—accounting for these differences may foster more appropriate comparisons between hospitals in analyses using a national sample. Although accounting for state Medicaid eligibility and enrollment factors appeared to have a negligible effect on overall hospital performance in our analysis, future work is encouraged to examine whether regional differences exist in uptake of Medicaid benefits among potentially eligible populations, and whether this influences disparity results.

Finally, these results further support ongoing efforts by hospitals to advance health equity by illuminating that disparities between DE and non-DE patients in most hospitals persist despite accounting for community-level characteristics. Hospitals may reduce these internal disparities by targeting data collection and analytical resources, cultural transformation efforts, and quality improvement activities on strengthening equity in outcomes.^30^ Even where hospital rankings in disparity performance were most altered by adjustment for community-level factors, the magnitude of change was relatively modest. We found that large, urban, non–safety net, academic teaching hospitals had the largest changes in within-hospital disparities after adjusting for community-level indicators of beneficiary social risk. This finding differs from prior work examining the association of patient socioeconomic characteristics with hospital readmission rates,^31,32^ and it may be explained by the relatively high prevalence of community factors among patients who are not DE in these most affected hospitals, where there were otherwise relatively low shares of DE patients for each study condition.

### Limitations

First, although we included a wide set of covariates covering state policy, health services, and social risk, there may be additional social or nonsocial risk factors related to readmission that we did not include. Varying concentrations of such risks among DE patients or within certain hospitals may still influence reported within-hospital disparities. Second, by accounting for social risk largely through community-level variables, our approach overlooks social risk heterogeneity within communities—though it is unclear in what direction such heterogeneity may bias results. An example of this is race, where we included community-level racial composition, limiting our ability to detect the effect on disparities of individual structural or societal pressures felt differentially by individuals of different races. Because Medicare claims contain limited patient-level social risk data, our decision to use community-level covariates sought to preserve the full cohorts used by CMS, rather than alternative smaller subcohorts with available patient-level social risk survey data.^23,33,34^ Third, although we examined community-level risks within ZCTAs, it is possible that use of more granular areas, such as census tracts, may have affected study findings, though prior related work demonstrated comparability in results when using zip-based measures of social risk compared with census tracts.^23,35^ Finally, we examined outcomes for older adults across 3 common conditions, and so the results may be less generalizable to younger DE patients who may have different health and functional needs, or patients hospitalized for other conditions where the drivers for readmission may be different.

## Conclusions

In this cohort study, within-hospital disparities in 30-day readmission for DE patients were only moderately explained by differences in social risk measured at the community level. This suggests that hospital efforts to advance equity should focus on improving the quality of care transitions at discharge for hospitalized DE patients.

## Notes

Supplement. eAppendix A. Summary of Within-Hospital Disparity Method eAppendix B. Estimates of Medicaid Enrollment by Eligibility Pathway for Older Adult Dual Eligible Medicare Beneficiaries eAppendix C. Additional analytic results
