# Supplementary material for: Factors Associated With Disparities in Hospital Readmission Rates Among US Adults Dually Eligible for Medicare and Medicaid
Source: JAMA Health Forum. 2022 Jan 28;3(1):e214611. doi: 10.1001/jamahealthforum.2021.4611 (PMC8903116; doi:10.1001/jamahealthforum.2021.4611)
Supplement: Supplement. — eAppendix A. Summary of Within-Hospital Disparity Method eAppendix B. Estimates of Medicaid Enrollment by Eligibility Pathway for Older Adult Dual Eligible Medicare Beneficiaries eAppendix C. Additional analytic results [file jamahealthforum-e214611-s001.pdf]

Supplemental Online Content

Silvestri D, Goutos D, Lloren A, et al. Factors associated with disparities in hospital readmission rates among US adults dually eligible for Medicare and Medicaid. *JAMA Health Forum*. 2022;3(1):e214611. doi:10.1001/jamahealthforum.2021.4611

eAppendix A. Summary of Within-Hospital Disparity Method
eAppendix B. Estimates of Medicaid Enrollment by Eligibility Pathway for Older Adult Dual Eligible Medicare Beneficiaries
eAppendix C. Additional analytic results

This supplemental material has been provided by the authors to give readers additional information about their work.

© 2022 Silvestri D, et al. *JAMA Health Forum.*

Appendix A: Summary of Within-Hospital Disparity Method

The Within-Hospital Disparity Method is an extension of the risk standardization approach developed and reported by CMS for dichotomous outcome measures, including readmission. The method was developed to assess the difference in risk-adjusted readmission rates between dual eligible and all other Medicare beneficiaries within a given hospital, though in theory the method could also be applied to any additional dichotomous social indicator of risk (beyond dual eligibility) or outcome measure (beyond readmission). The within-hospital disparity model, as an extension of CMS' underlying risk-adjustment model, is summarized below:

Underlying Risk Adjustment Model

Suppose Y_{ij} indicates whether the ith patient at the jth hospital is readmitted within 30 days, and $\mathbf{Z_{ij}}$ is a vector of risk factors for that patient. Then, we would first estimate a mixed effects model:

$$\text{logit}(\Pr[Y_{ij} = 1]) = \beta_0 + \boldsymbol{B}^T \mathbf{Z_{ij}} + \gamma_j \qquad (1)$$

$$\gamma_j \sim N(0, \tau^2)$$

where γ_j is a random hospital effect. The random effect γ_j , sometimes called the "hospital-specific effect," can be interpreted as a latent quality trait for hospital j because it estimates the contribution of the hospital to the outcome risk for all patients admitted to hospital j. Once model (1) is estimated, it is used for these measures to calculate for each patient a predicted probability of the outcome P_{ij} and an expected probability E_{ij} where

$$P_{ij} = \text{logit}^{-1}(\beta_0 + \boldsymbol{B}^T \mathbf{Z_{ij}} + \gamma_j); \qquad E_{ij} = \text{logit}^{-1}(\beta_0 + \boldsymbol{B}^T \mathbf{Z_{ij}})$$

These represent the predicted risk for patient i using hospital j's specific latent quality and the risk predicted for the same patient assuming he or she were treated at a hospital with average latent quality. Once these are calculated, they are used to construct a standardized risk ratio (SRR) for each hospital j:

$$SRR_j = (\Sigma P_{ij})/(\Sigma E_{ij}) \qquad (2)$$

where the sum is over all patients at hospital j. This is usually multiplied by the overall crude rate mean (Y_{ij}) to produce a risk-standardized readmission rate (RSRR), which is reported.

Disparity Model

Model (1) can be expanded to include an additional risk factor X (for example, dual eligibility), which captures the fixed effect of X on patient outcomes:

$$\text{logit}(\Pr[Y_{ij} = 1]) = \beta_0 + \mathbf{BZ_{ij}} + \beta_X X_{ij} + \gamma_j \qquad (3)$$

$$\gamma_j \sim N(0, \tau^2)$$

Here, β_X represents the *overall disparity effect*. While important to assess, it is a fixed effect, which is the same for all hospitals. To assess within-hospital disparities related to patient attribute X (for example, dual eligibility), we assume that in addition to the hospital-specific effect described above and the fixed effect $\beta_{X,}$ there is an additional latent disparity trait at each hospital, such that patients with X=1 have an increased or decreased risk of the outcome specific to that hospital:

$$\text{logit}(\Pr[Y_{ij} = 1]) = \beta_0 + \mathbf{BZ_{ij}} + (\beta_X + \varepsilon_j) X_{ij} + \gamma_j \qquad (4)$$

$$(\gamma_j , \varepsilon_j) \sim N(0, \Sigma^2)$$

where ε_j is the *hospital-specific disparity effect* (or within-hospital disparity effect), and represents the latent disparity trait for each hospital. Model (4) is known as a "mixed effects random slope model". There are different ways of specifying the same model, but for purposes of estimation we use a form that separates the between-hospital effect (effect of being at a hospital with a high proportion of patients with the risk factor) from the within-hospital effect (effect of having the social risk factor at a particular hospital). In order to better interpret the results, we also center all factors $\mathbf{Z}_{ij}$ on their overall mean. Thus, our final model is:

$$\text{logit}(\Pr[\text{Yij } = 1])\,) = \beta_0 + \beta_1\left(Z_{ij1} - Z_{..1}\right) + \cdots + \beta_p\left(Z_{ijp} - Z_{..p}\right) + \gamma_j + \beta_x\left(X_{ij} - X_{j\cdot}\right)$$
$$+\beta_{x2}\left(X_{j\cdot} - X_{..}\right) + \epsilon_j\left(X_{ij} - X_{j\cdot}\right) \qquad (5)$$

where

- $Z_{..k} = \frac{1}{\sum_{i=1}^{I} n_i} \sum_{i=1}^{I} \sum_{j=1}^{n_i} Z_{ijk}$ for k=1, … , p;
- X_{ij} is the indicator of social risk factor (for example, 1=dual, 0=non-dual or 1=Black, 0=White) for case i at hospital *j*;
- $X_{j\cdot} = \frac{1}{n_j} \sum_{i=1}^{n_j} X_{ij}$ is the proportion of cases with social risk factors in hospital j and $X_{..} = \frac{1}{I} \sum_{j=1}^{I} X_{j\cdot}$ is the average of all hospitals proportion of cases with social risk factors;
- $(\gamma_j, \epsilon_j)' \sim \text{N}_2(0, \Sigma)$ with $\Sigma = \begin{pmatrix} \sigma_0^2 & \sigma_{01} \\ \sigma_{01} & \sigma_1^2 \end{pmatrix}$.

In this model, the fixed effect β_x reflects overall disparity; that is, the average disparity effect across all hospitals. The random slope ϵ_j reflects hospital i's hospital-specific disparity effect; that is the degree to which the disparity in outcomes in hospital j differs from the average disparity. By combining these two, we can estimate the disparity effect at a given hospital.

Reporting

Once model (5) is estimated, we report the hospital disparity using in a metric that is both accurate and accessible to consumers: the rate difference (RD). The rate difference is calculated from model (5) by predicting the probability of a positive outcome under two different assumptions and calculating the difference. In both cases, we assume **Z=**mean**(**$\mathbf{Z}_{ij}$**)**, the average value of all risk factors in the population, and include the hospital-specific quality effect γ_j and hospital-specific disparity ε_j. For one, we assume X_{ij}= 0, that the hypothetical patient has no disparity risk factor, and for the other we assume X_{ij}=1, that the hypothetical average patient has the disparity risk factor. The difference between these two predicted probabilities is the rate difference, which can be intuitively interpreted as the difference in outcome rates for "average patients" treated at that hospital with and without the social risk factor.

© 2022 Silvestri D, et al. *JAMA Health Forum.*

Appendix B: Estimates of Medicaid Enrollment by Eligibility Pathway for Older Adult Dual Eligible Medicare Beneficiaries

We categorized states based on Medicaid income or asset eligibility pathways for older adults (>65 years). Using publicly available data sources[1,2,3], supplemented by manual review of state Medicaid websites, we characterized state income and asset thresholds for two eligibility groups, those that are categorically eligible by income or asset level (eTable 1, columns B and C) and those eligible through medically-needy determinations (eTable 1, columns D and E). We also categorized states based on the type of authorities used for establishing enrollment, resulting in three groups: those that use a single federal application and federal criteria (Section 1634), those that use a separate state application and federal criteria (SSI Criteria), and those that use state-specific application and criteria (Section 209b) (eTable 1, Column F). An entry of "N/A" signifies that the state does not participate in the eligibility pathway.

eTable 1

(A)	(B)	(C)	(D)	(E)	(F)
**State**	**Categorical/Poverty (Income as % FPL)**	**Categorical/Poverty (Asset)**	**Medically Needy (Income as % FPL)**	**Medically Needy (Asset)**	**Enrollment Authority**
AL	73%	$2,000	N/A	N/A	1634
AK	59%	$2,000	N/A	N/A	SSI Criteria
AZ	100%	$10,000	N/A	N/A	1634
AR	80%	$7,280	11%	$2,000	1634
CA	100%	$2,000	60%	$2,000	1634
CO	73%	$2,000	N/A	N/A	1634
CT	63%	$1,600	63%	$1,600	209(B)
DC	100%	$4,000	64%	$4,000	1634
DE	73%	$2,000	N/A	N/A	1634
FL	88%	$5,000	18%	$5,000	1634
GA	73%	$2,000	32%	$2,000	1634
HI	100%	$2,000	41%	$2,000	209(B)
ID	76%	$2,000	N/A	N/A	SSI Criteria
IL	100%	$2,000	100%	$2,000	209(B)
IN	73%	$2,000	N/A	N/A	1634
IA	73%	$2,000	48%	$10,000	1634
KS	73%	$2,000	47%	$2,000	SSI Criteria
KY	73%	$2,000	22%	$2,000	1634
LA	73%	$2,000	10%	$2,000	1634
ME	100%	$2,000	31%	$2,000	1634
MD	73%	$2,000	35%	$2,000	1634
MA	100%	$2,000	52%	$2,000	1634
MI	100%	$2,000	41%	$2,000	1634
MN	100%	$3,000	80%	$3,000	209(B)
MS	73%	$2,000	N/A	N/A	1634
MO	85%	$1,000	85%	$2,000	209(B)
MT	73%	$2,000	52%	$2,000	1634

© 2022 Silvestri D, et al. *JAMA Health Forum.*

NE	100%	$4,000	39%	$4,000	SSI Criteria
NV	73%	$2,000	N/A	N/A	SSI Criteria
NH	75%	$1,500	59%	$2,500	209(B)
NJ	100%	$4,000	37%	$4,000	1634
NM	73%	$2,000	N/A	N/A	1634
NY	82%	$2,000	82%	$14,850	1634
NC	100%	$2,000	24%	$2,000	1634
ND	83%	$3,000	83%	$3,000	209(B)
OH	73%	$1,500	N/A	N/A	1634
OK	100%	$2,000	N/A	N/A	SSI Criteria
OR	73%	$2,000	N/A	N/A	SSI Criteria
PA	100%	$2,000	42%	$2,400	1634
RI	100%	$4,000	88%	$4,000	1634
SC	100%	$7,280	N/A	N/A	1634
SD	73%	$2,000	N/A	N/A	1634
TN	73%	$2,000	N/A	N/A	1634
TX	73%	$2,000	N/A	N/A	1634
UT	100%	$2,000	100%	$2,000	SSI Criteria
VT	73%	$2,000	110%	$2,000	1634
VA	80%	$2,000	46%	$2,000	209(B)
WA	73%	$2,000	73%	$2,000	1634
WV	73%	$2,000	20%	$2,000	1634
WI	81%	$2,000	59%	$2,000	1634
WY	73%	$2,000	N/A	N/A	1634

To assess the validity of this approach, we used the 2012 Medicaid Analytic eXtract (MAX) dataset to examine enrollment differences across these two eligibility groups. MAX contains patient-level demographic, enrollment, and utilization information obtained from the State Medicaid Statistical Information System. We selected the 2012 version of the dataset as it contains the most comprehensive state-level information available.

We included 45 states and DC in this analysis. We excluded three states (Colorado, Rhode Island, and Kansas) for which data were unavailable in our version of the dataset at the time of analysis and two additional states (Idaho and Missouri) where anomalies in dual eligibility reporting have been previously identified in the 2012 MAX files. Collectively, the excluded states account for only 5% of the total U.S. population.

We used the Medicare crossover variable to categorize beneficiaries into (a) full benefit dual eligible and (b) all other beneficiaries. This information uses data derived from the Medicare Modernization Act information submitted to CMS by each State Medicaid Agency, and is considered the gold standard for identifying dual eligible enrollees in Medicare data.

© 2022 Silvestri D, et al. *JAMA Health Forum.*

The Medicaid eligibility pathway and type of dual eligibility were obtained using the most recent monthly enrollment indicator available for the beneficiary. We excluded individuals with more than a two-month gap in enrollment.

In total, we identified 3.4 million beneficiaries 65 years and older as of January 1, 2012 who received full Medicaid and Medicare benefits and met inclusion criteria. We categorized these individuals into mutually exclusive groupings based on their eligibility pathway: (a) categorically eligible/poverty level, (b) medically needy, and (c) other (including all other pathways). For the purposes of data presentation, we combined aged, blind, and disabled individuals into one group, given that we limited the cohort to 65 years and older.

The figure below depicts the cumulative percentage of older adult dual eligible population by eligibility category. The darker the shading of blue, the more the eligibility pathway accounts for the total dual eligible population.

The figure shows that nationally:

- Two-thirds (66.2%) of older adults who qualify for full Medicaid and Medicare benefits qualified for Medicaid benefits as a result of receiving SSI benefits or meeting state-specific 209(b) eligibility thresholds ("categorically eligible") or resided in a state that expanded the categorically eligible group to allow income levels up to the federal poverty level.
- Thirteen percent (13.0%) of older adults who qualify for full Medicaid and Medicare benefits received Medicaid benefits under the medically needy ("spend down") pathway.

Eligibility Pathways and Variation in State Policies for Older Adults with Full Medicaid and Medicare Benefits

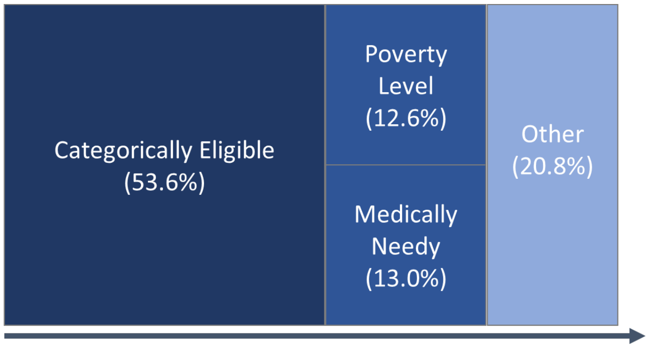

References (*Appendix B*)

1. Roberts ET, Mellor JM, McInerney M, Sabik LM. State variation in the characteristics of Medicare-Medicaid dual enrollees: Implications for risk adjustment. *Health Serv Res.* 2019;54(6):1233-1245.
2. Brown JR, Chang CH, Zhou W, MacKenzie TA, Malenka DJ, Goodman DC. Health system characteristics and rates of readmission after acute myocardial infarction in the United States. *J Am Heart Assoc.* 2014;3(3):e000714.
3. Watts MOM, Cornachione E, Musumeci M. Medicaid Financial Eligibility for Seniors and People with Disabilities in 2015*. The Henry J. Kaiser Family Foundation*; 2016.

© 2022 Silvestri D, et al. *JAMA Health Forum.*

Appendix C: Additional Analytic Results

eTable 2: Median hospital prevalence of community-level indicators of social risk among all Medicare beneficiaries, by degree of hospital change in within-hospital disparity after adjustment for state Medicaid policies, local health service availability, and community-level indicators of social risk

	Acute Myocardial Infarction (AMI)			Heart Failure (HF)			Pneumonia		
	Median hospital prevalence (IQR)[1]			Median hospital prevalence (IQR)[1]			Median hospital prevalence (IQR)[1]		
	Hospitals with high change in disparity[2]	Hospitals with low change in disparity[2]	*p*[3]	Hospitals with high change in disparity[2]	Hospitals with low change in disparity[2]	*p*[3]	Hospitals with high change in disparity[2]	Hospitals with low change in disparity[2]	*p*[3]
**Socioeconomic position**									
Low composite SES score[4]	13.5% (7.0-20.9)	11.1% (0.0-25.6)	0.005	20.8% (10.4-33.6)	12.8% (3.3-28.4)	<0.001	18.9% (10.9-30.7)	12.3% (3.7-26.2)	<0.001
**Race, Ethnicity, Cultural Context**									
Black race[4]	7.8% (1.5-25.0)	2.1% (0.0-33.3)	<0.001	38.3% (15.5-68.2)	2.2% (0.0-32.8)	<0.001	35.8% (18.8-70.8)	2.4% (0.0-29.7)	<0.001
Hispanic ethnicity[4]	13.8% (1.7-44.1)	2.8% (0.0-45.5)	<0.001	22.6% (3.2-63.6)	3.5% (0.0-43.5)	<0.001	33.3% (4.8-66.1)	3.4% (0.6-42.0)	<0.001
Limited English proficiency[4]	26.3% (4.3-60.3)	8.5% (0.0-50.9)	<0.001	36.5% (10.4-81.2)	7.8% (1.0-51.7)	<0.001	49.7% (13.0-86.3)	7.7% (1.6-51.2)	<0.001
Non-US born[4]	25.0% (3.1-66.2)	5.7% (0.0-52.1)	<0.001	47.4% (13.1-94.5)	5.1% (0.4-48.6)	<0.001	63.9% (16.4-96.0)	5.3% (1.3-48.6)	<0.001
**Social Relationships**									
Unmarried or spouse absent[4]	22.6% (9.6-35.3)	9.1% (0.0-32.7)	<0.001	41.3% (23.8-61.4)	8.7% (1.3-32.5)	<0.001	38.9% (22.9-57.8)	8.8% (2.0-32.0)	<0.001
Living without family[4]	17.7% (8.3-28.1)	10.1% (0.0-30.4)	<0.001	26.3% (9.4-41.8)	10.8% (2.9-30.8)	<0.001	23.2% (10.2-41.2)	11.3% (3.6-30.8)	<0.001
**Residential/Community Context**									
Poor vehicular availability[4]	23.8% (11.0-36.3)	13.6% (0.0-40.0)	<0.001	43.7% (26.2-69.4)	13.0% (2.3-38.9)	<0.001	44.7% (26.6-71.7)	12.6% (2.9-37.9)	<0.001
Vacant housing[4]	3.1% (0.9-6.6)	1.3% (0.0-9.0)	<0.001	2.1% (0.6-6.7)	2.6% (0.2-9.8)	0.438	2.6% (0.7-6.6)	2.7% (0.7-9.6)	0.318
Food or cash assistance[4]	20.8% (10.1-32.5)	15.5% (0.0-36.8)	0.005	18.7% (4.1-34.5)	16.4% (4.5-37.3)	0.934	19.2% (4.0-32.5)	16.1% (5.3-35.7)	0.479

1: Hospital prevalence is among hospitalizations within the corresponding condition-specific cohort within each hospital (AMI: 507,219 hospitalizations within 4,188 hospitals; HF: 1,214,282 hospitalizations within 4,697 hospitals; Pneumonia: 1,419,412 hospitalizations within 4,749 hospitals)
2: Within each condition-specific cohort, hospitals with high changes in disparities are the top 5% of hospitals in terms of the change in within-hospital disparities after adjusting for state-level dual eligibility policies, county-level health service availability, and community-level indicators of social risk. Hospitals with low changes in disparities reflect the remaining 95%.
3: p-values for each variable reflect the difference in median hospital variable prevalence between hospitals with low and high within-hospital disparities, and are computed using Kruskal-Wallis test for comparison using alpha=0.05.

© 2022 Silvestri D, et al. *JAMA Health Forum.*

4: Variables indicate beneficiary residence in the ZCTA with the highest-risk quintile in terms of AHRQ SES index composite score (lowest), black race (highest), Hispanic origin (highest), percent of population 5 years or older who do not speak English at home and who speak English less than “very well” (highest), percent of population who are non-US born (highest), percent of population 15 years or older who are unmarried or married with spouse absent (highest), percent of population 65 years or older who live in non-family or group homes (highest), percent of households with no vehicular availability (highest), percent of housing units that are vacant (highest), or percent of population receiving food or cash assistance (highest). Reference is the remaining four quintiles.

© 2022 Silvestri D, et al. *JAMA Health Forum.*

eTable 3: Median hospital prevalence of community-level indicators of social risk among dual eligible Medicare beneficiaries, by degree of hospital change in within-hospital disparity after adjustment for state Medicaid policies, local health service availability, and community-level indicators of social risk

	Acute Myocardial Infarction (AMI)			Heart Failure (HF)			Pneumonia		
	Median hospital prevalence (IQR)[1]			Median hospital prevalence (IQR)[1]			Median hospital prevalence (IQR)[1]		
	Hospitals with high change in disparity[2]	Hospitals with low change in disparity[2]	p^3	Hospitals with high change in disparity[2]	Hospitals with low change in disparity[2]	p^3	Hospitals with high change in disparity[2]	Hospitals with low change in disparity[2]	p^3
**Socioeconomic position**									
Low composite SES score[4]	28.1% (13.6-42.3)	20.0% (0.0-43.5)	<0.001	35.8% (21.7-50.0)	21.4% (1.8-40.5)	<0.001	30.4% (19.0-43.9)	18.8% (4.8-35.8)	<0.001
**Race, Ethnicity, Cultural Context**									
Black race[4]	12.5% (0.0-43.8)	1.7% (0.0-50.0)	0.002	54.7% (24.4-81.4)	2.0% (0.0-42.5)	<0.001	47.5% (27.8-78.5)	2.9% (0.0-37.5)	<0.001
Hispanic ethnicity[4]	20.8% (1.2-67.7)	0.0% (0.0-54.5)	<0.001	29.3% (3.0-68.9)	2.4% (0.0-50.0)	<0.001	35.4% (5.6-71.4)	3.8% (0.0-48.0)	<0.001
Limited English proficiency[4]	41.4% (4.5-80.2)	8.7% (0.0-66.7)	<0.001	46.4% (13.9-90.3)	7.7% (0.0-60.0)	<0.001	58.1% (15.7-91.6)	8.8% (0.0-58.5)	<0.001
Non-US born[4]	35.6% (4.3-80.0)	4.5% (0.0-66.7)	<0.001	52.7% (13.3-94.4)	4.2 (0.0-52.6)	<0.001	70.3% (20.3-97.5)	5.9% (0.0-51.5)	<0.001
**Social Relationships**									
Unmarried or spouse absent[4]	36.4% (12.5-58.8)	14.3% (0.0-48.8)	<0.001	57.6% (36.2-78.3)	12.3% (0.0-44.7)	<0.001	52.3% (33.3-71.2)	12.5% (0.0-42.1)	<0.001
Living without family[4]	25.0% (11.1-43.2)	11.1% (0.0-37.2)	<0.001	32.1% (13.3-53.8)	12.5% (0.0-37.4)	<0.001	31.6% (13.1-51.0)	14.2% (3.0-38.3)	<0.001
**Residential/Community Context**									
Poor vehicular availability[4]	39.0% (17.0-60.0)	21.1% (0.0-50.0)	<0.001	61.6% (34.7-84.5)	18.8% (0.0-50.0)	<0.001	57.5% (38.4-83.9)	17.1% (2.6-47.7)	<0.001
Vacant housing[4]	0.0% (0.0-5.6)	0.0% (0.0-6.3)	0.024	1.2% (0.0-6.8)	0.0% (0.0-8.3)	0.050	1.5% (0.0-5.4)	1.4% (0.0-8.9)	0.565
Food or cash assistance[4]	18.7% (2.9-35.7)	13.0% (0.0-41.3)	0.051	18.9% (2.6-36.5)	14.7% (0.0-40.0)	0.338	16.7% (2.5-35.7)	15.4% (3.2-40.0)	0.835

1: Hospital prevalence is among hospitalizations within the corresponding condition-specific cohort within each hospital (AMI: 66,201 hospitalizations within 3,491 hospitals; HF: 208,311 hospitalizations within 4,442 hospitals; Pneumonia: 321,506 hospitalizations within 4,617 hospitals)
2: Within each condition-specific cohort, hospitals with high changes in disparities are the top 5% of hospitals in terms of the change in within-hospital disparities after adjusting for state-level dual eligibility policies, county-level health service availability, and community-level indicators of social risk. Hospitals with low changes in disparities reflect the remaining 95%.
3: p-values for each variable reflect the difference in median hospital variable prevalence between hospitals with low and high within-hospital disparities, and are computed using Kruskal-Wallis test for comparison using alpha=0.05.
4: Variables indicate beneficiary residence in the ZCTA with the highest-risk quintile in terms of AHRQ SES index composite score (lowest), black race (highest), Hispanic origin (highest), percent of population 5 years or older who do not speak English at home and

© 2022 Silvestri D, et al. *JAMA Health Forum.*

who speak English less than “very well” (highest), percent of population who are non-US born (highest), percent of population 15 years or older who are unmarried or married with spouse absent (highest), percent of population 65 years or older who live in non-family or group homes (highest), percent of households with no vehicular availability (highest), percent of housing units that are vacant (highest), or percent of population receiving food or cash assistance (highest). Reference is the remaining four quintiles.

© 2022 Silvestri D, et al. *JAMA Health Forum.*

eTable 4: Median hospital prevalence of community-level indicators of social risk among Medicare beneficiaries not dual eligible for Medicaid, by degree of hospital change in within-hospital disparity after adjustment for state Medicaid policies, local health service availability, and community-level indicators of social risk

	Acute Myocardial Infarction (AMI)			Heart Failure (HF)			Pneumonia		
	Median hospital prevalence (IQR)[1]			Median hospital prevalence (IQR)[1]			Median hospital prevalence (IQR)[1]		
	Hospitals with high change in disparity[2]	Hospitals with low change in disparity[2]	*p*[3]	Hospitals with high change in disparity[2]	Hospitals with low change in disparity[2]	*p*[3]	Hospitals with high change in disparity[2]	Hospitals with low change in disparity[2]	*p*[3]
**Socioeconomic position**									
Low composite SES score[4]	13.5% (7.0-20.9)	11.1% (0.0-25.6)	<0.001	20.8% (10.4-33.6)	12.8% (3.3-28.4)	<0.001	18.9% (10.9-30.7)	12.3% (3.7-26.2)	<0.001
**Race, Ethnicity, Cultural Context**									
Black race[4]	7.8% (1.5-25.0)	2.1% (0.0-33.3)	<0.001	38.3% (15.5-68.2)	2.2% (0.0-32.8)	<0.001	35.8% (18.8-70.8)	2.4% (0.0-29.7)	<0.001
Hispanic ethnicity[4]	13.8% (1.7-44.1)	2.8% (0.0-45.5)	<0.001	22.6% (3.2-63.6)	3.5% (0.0-43.5)	<0.001	33.3% (4.8-66.1)	3.4% (0.6-42.0)	<0.001
Limited English proficiency[4]	26.3% (4.3-60.3)	8.5% (0.0-50.9)	<0.001	36.5% (10.4-81.2)	7.8% (1.0-51.7)	<0.001	49.7% (13.0-86.3)	7.7% (1.6-51.2)	<0.001
Non-US born[4]	25.0% (3.1-66.2)	5.7% (0.0-52.1)	<0.001	47.4% (13.1-94.5)	5.1% (0.4-48.6)	<0.001	63.9% (16.4-96.0)	5.3% (1.3-48.6)	<0.001
**Social Relationships**									
Unmarried or spouse absent[4]	22.6% (9.6-35.3)	9.1% (0.0-32.7)	<0.001	41.3% (23.8-61.4)	8.7% (1.3-32.5)	<0.001	38.9% (22.9-57.8)	8.8% (2.0-32.0)	<0.001
Living without family[4]	17.7% (8.3-28.1)	10.1% (0.0-30.4)	<0.001	26.3% (9.4-41.8)	10.8% (2.9-30.8)	<0.001	23.2% (10.2-41.2)	11.3% (3.6-30.8)	<0.001
**Residential/Community Context**									
Poor vehicular availability[4]	23.8% (11.0-36.3)	13.6% (0.0-40.0)	<0.001	43.7% (26.2-69.4)	13.0% (2.3-38.9)	<0.001	44.7% (26.6-71.7)	12.6% (2.9-37.9)	<0.001
Vacant housing[4]	3.1% (0.9-6.6)	1.3% (0.0-9.0)	<0.001	2.1% (0.6-6.7)	2.6% (0.2-9.8)	0.438	2.6% (0.7-6.6)	2.7% (0.7-9.6)	<0.001
Food or cash assistance[4]	20.8% (10.1-32.5)	15.5% (0.0-36.8)	0.005	18.7% (4.1-34.5)	16.4% (4.5-37.3)	0.934	19.2% (4.0-32.5)	16.1% (5.3-35.7)	0.479

1: Hospital prevalence is among hospitalizations within the corresponding condition-specific cohort within each hospital (AMI: 434,072 hospitalizations within 4,064 hospitals; HF: 989,223 hospitalizations within 4,671 hospitals; Pneumonia: 1,078,693 hospitalizations within 4,724 hospitals)
2: Within each condition-specific cohort, hospitals with high changes in disparities are the top 5% of hospitals in terms of the change in within-hospital disparities after adjusting for state-level dual eligibility policies, county-level health service availability, and community-level indicators of social risk. Hospitals with low changes in disparities reflect the remaining 95%.
3: p-values for each variable reflect the difference in median hospital variable prevalence between hospitals with low and high within-hospital disparities, and are computed using Kruskal-Wallis test for comparison using alpha=0.05.

© 2022 Silvestri D, et al. *JAMA Health Forum.*

4: Variables indicate beneficiary residence in the ZCTA with the highest-risk quintile in terms of AHRQ SES index composite score (lowest), black race (highest), Hispanic origin (highest), percent of population 5 years or older who do not speak English at home and who speak English less than "very well" (highest), percent of population who are non-US born (highest), percent of population 15 years or older who are unmarried or married with spouse absent (highest), percent of population 65 years or older who live in non-family or group homes (highest), percent of households with no vehicular availability (highest), percent of housing units that are vacant (highest), or percent of population receiving food or cash assistance (highest). Reference is the remaining four quintiles.

© 2022 Silvestri D, et al. *JAMA Health Forum.*
